# Emotional state of healthcare workers in hospital red-zone during COVID-19 Pandemic

**DOI:** 10.1192/j.eurpsy.2022.967

**Published:** 2022-09-01

**Authors:** S. Kryzhanovskiy, M. Samushia, T. Berishvili, V. Shmirev, N. Amosova

**Affiliations:** 1 Central State Medical Academy of Department of Presidential Affairs Moscow, Russia, Scientific Department, Moscow, Russian Federation; 2 Central State Medical Academy of Department of Presidential Affairs Moscow, Russia, Scientific Department, moscow, Russian Federation

**Keywords:** healthcare, Covid-19, workers, red-zone

## Abstract

**Introduction:**

The COVID-19 pandemic has a significant impact on the mental state of not only quarantined citizens and patients, but also health workers.

**Objectives:**

The aim of the study was to assess of the mental health of doctors involved in work in the “red zone” during the COVID-19 pandemic.

**Methods:**

77 respondents were interviewed using the HADS questionnaires and the Maslach burnout test. For statistical data processing Microsoft Office Excel 2016, IBM SPSS were used.

**Results:**

An increase on the depression scales was noted in 7%, anxiety in 23%, and anxiety and depression together 27%. According to the Maslach questionnaire, 32 (41.5%) doctors noted a reaction of the type of “emotional devastation”. 10 (12.9%) doctors noted a reaction “reduction of professional achievements”. Three doctors (3.8%) had a dehumanization reaction in the form of dull emotions to colleagues and patients.

**Conclusions:**

Work in the “red zone” has a significant negative impact on the mental health of doctors and medical personnel

**Disclosure:**

No significant relationships.

